# The Effectiveness of Exercise in Reducing Cardiovascular Risk Factors Among Adults: A Systematic Review and Meta-Analysis

**DOI:** 10.7759/cureus.68928

**Published:** 2024-09-08

**Authors:** Mohd Diya Masmoum, Soha Khan, Waqas A. Usmani, Raheel Chaudhry, Rubela Ray, Arhum Mahmood, Maheen Afzal, Muhammad Sohail S Mirza

**Affiliations:** 1 General Practice, Alfaisal University College of Medicine, Riyadh, SAU; 2 Medicine, Fatima Jinnah Medical University, Lahore, PAK; 3 Medicine, Ziauddin University, Karachi, PAK; 4 Medicine, Baylor College of Medicine, Houston, USA; 5 Internal Medicine, Bankura Sammilani Medical College and Hospital, Bankura, IND; 6 Internal Medicine, Henry Ford Health System, Detroit, USA; 7 Medical Office, Allama Iqbal Medical College, Lahore, PAK; 8 Internal Medicine, Shandong University School of Medicine, Jinan, CHN

**Keywords:** blood pressure, body mass, cardiovascular, cardiovascular risk factors, cholesterol, excercise

## Abstract

Cardiovascular disease (CVD) remains one of the major causes of sickness and death in the world. However, lifestyle modifications, such as exercise, can significantly reduce the risk of this disease. This study aimed to assess the effectiveness of various forms of physical activity in reducing CVD risk factors among adults.

A comprehensive search of the databases PubMed, Cumulative Index to Nursing and Allied Health Literature (CINAHL), Cochrane Central Register of Controlled Trials (CENTRAL), and Excerpta Medica Database (EMBASE) databases was conducted between January 1, 2014, and May 31, 2024, as per Preferred Reporting Items for Systematic Reviews and Meta-Analyses (PRISMA) guidelines. Randomized controlled trials (RCTs), cohort studies, and observational studies on the impact of aerobic, resistance, or combined training on cardiovascular risk factors in adults (≥18 years) were considered for inclusion. Data relating to primary outcomes, including stroke and myocardial infarction rates, BP, cholesterol levels, and BMI were collected. The Cochrane risk-of-bias tool and the Methodological Index for Non-Randomized Studies (MINORS) checklist were used for quality and bias assessment. Meta-analyses were performed using the RevMan software, with heterogeneity evaluated by I² statistics; 17 studies, including 11 RCTs and six cohort studies, met the inclusion criteria.

There was a significant reduction in the mean systolic BP (SBP) by 3.32 mmHg [95% confidence interval (CI): 0.85-5.78 mmHg; p<0.0001] and mean diastolic BP (DBP) by 2.99 mmHg (95% CI: 2.34-3.64 mmHg; p < 0.00001) after exercise interventions. Moreover, cholesterol levels and BMI values improved with exercise. Those who exercised had a lower risk of stroke or heart attack compared with the controls [odds ratio (OR): 0.57; 95% CI: 0.28-1.14; p >0.0001], although there was substantial heterogeneity in effect size across the studies (I² = 98%).

Different types of physical activity (i.e., aerobic, resistance, or combined exercise) can effectively reduce key cardiovascular risk factors, including BP, cholesterol levels, and BMI values. Regular physical activity is still regarded as the most effective preventive measure against CVD, despite inconsistencies in research findings. Future studies should aim to identify optimal exercise programs and their long-term effects on diverse populations.

## Introduction and background

Cardiovascular disease (CVD) remains one of the main causes of morbidity and mortality globally, with an estimated 17.9 million deaths per year, accounting for 31% of the total number of deaths across the globe [1]. Despite considerable advancements in medication therapies, lifestyle modifications remain the most effective approach to managing cardiovascular risks, with exercise standing out as a key factor in mitigating CVD risks while promoting overall heart health [2]. Exercise generally involves various types of physical activity, including endurance training (e.g., running, walking, and cycling) and resistance training (e.g., weightlifting). Regular physical activity improves cardiovascular health via multiple mechanisms, such as improving lipid metabolism, managing hypertension through reduced blood volume, enhancing glucose metabolism, and preventing weight gain [3]. Also, exercise positively impacts vascular performance, inflammation, and oxidative distress, highlighting that these are all crucial elements in CVD pathogenesis [4].

Exercise employs multiple mechanisms that benefit the heart and blood vessels. For example, aerobic exercise boosts endothelial function by increasing nitric oxide bioavailability, thus leading to vasodilation and decreased arterial stiffness [5]. Moreover, regular workouts have been proven to modulate autonomic nerve balance, decreasing sympathetic activity and promoting parasympathetic tone, which, in turn, helps lower BP and resting heart rate to some extent [6]. Physical activity can also help fight systemic inflammation and improve lipid metabolism by reducing pro-inflammatory markers such as C-reactive protein (CRP) while increasing beneficial cholesterol levels [7].

Prior meta-analyses have highlighted the advantages of physical exercise for cardiovascular function; however, wide variations in effect size have been observed depending on the exercise types studied. For example, in a meta-analysis by Kodama et al. [8], aerobic exercise was demonstrated to reduce BP and improve lipid profiles, whereas Cornelissen and Fagard [9] identified resistance training as a factor contributing to cardiovascular risk reduction. Hence, given the diversity of exercise interventions and the rapidly evolving nature of exercise science, there is a need for a comprehensive, up-to-date meta-analysis that can integrate the latest evidence and examine different CVD risk factors and how they may be affected by various types of exercise.

This meta-analysis aims to assess the effectiveness of various physical exercise modalities in lowering CVD risks among adults. Specifically, the review (1) evaluates the effects of aerobic, resistance, and combined exercise on cardiovascular outcomes, including BP, lipid profiles, glucose metabolism, and inflammatory markers; (2) endeavors to determine to what extent exercise interventions affect cardiovascular hazard decrement; (3) aims to identify potential moderators of exercise efficacy (e.g., exercise intensity, duration, and frequency), as well as participant characteristics (e.g., sex, age, and baseline cardiovascular risk); and (4) provides evidence-based recommendations for incorporating physical activity into CVD prevention programs and management strategies.

## Review

Methodology

To conduct an up-to-date review and meta-analysis, we followed the Preferred Reporting Items for Systematic Reviews and Meta-Analyses (PRISMA) guidelines [10]. This research did not require additional ethical approval as all data were collected from published trials.

Data Sources and Search Terms

The search strategy for this systematic review and meta-analysis was designed to comprehensively identify studies evaluating the effectiveness of exercise in reducing cardiovascular risks among adults. The search was conducted using the electronic databases PubMed, CINAHL, Cochrane Central Register of Controlled Trials (CENTRAL), and EMBASE, covering the period from January 1, 2014, through May 31, 2024. The following search syntax, consisting of a combination of relevant keywords and Medical Subject Headings (MeSH) terms, was used: ((“exercise” OR “aerobic exercise” OR “resistance training” OR “mixed exercise training”) AND (“efficacy” OR “efficiency” OR “effectiveness” OR “roles”) AND (“CVD” OR “coronary heart disease” OR “cardiovascular risks” OR “heart disease risk”) AND (“general population” OR “adults” OR “older adults”)).

Study Selection and Eligibility Criteria

The present study followed the Population, Intervention, Comparison, and Outcomes (PICO) model, as shown in Table 1.

**Table 1:** PICO-based inclusion and exclusion criteria for study screening and selection PICO: Population, Intervention, Comparison, and Outcomes

Study characteristics	Criteria
Population	Adults aged 18 years and older with or without existing cardiovascular conditions; diverse demographics and geographical locations
Intervention	Structured exercise programs (aerobic, resistance, mixed); supervised and unsupervised exercise regimens; various exercise intensities and frequencies
Comparison	No exercise or usual care/control group; alternative interventions (e.g., dietary changes, medication); different types or intensities of exercise
Outcomes	Primary: reduction in cardiovascular events (heart attack, stroke); secondary: changes in cardiovascular risk factors (blood pressure, cholesterol levels, body mass index)
Design	Randomized controlled trials; longitudinal cohort studies; other observational studies (case-control, cross-sectional)

Studies were included in this meta-analysis if they (1) involved adults (18 years and older) either at risk for heart disease or not; (2) focused on exercise interventions, including aerobic, resistance, or combined training; (3) reported on CVD risk factors, such as BP, cholesterol levels, and BMI; (4) were randomized controlled trials (RCTs), cohorts, or of other observational designs; and (5) were accessible in full text and published in English.

Studies were excluded if they (1) examined groups with diabetes, obesity, polycystic ovary syndrome (PCOS), or other medical conditions; (2) utilized alternative therapeutic approaches, such as medications and nutritional modifications, instead of exercise training; (3) focused on outcomes rather than the frequency of heart attacks and strokes; (4) were systematic reviews, meta-analyses, scoping reviews, literature reviews, conferences, or letters that had already been published; or (5) were non-full-text articles or duplicate publications in languages other than English.

Data Extraction

After screening and selecting research articles, we extracted the following data from each eligible paper: author(s), study year, country, study design, study population, exercise type, and primary outcomes (e.g., stroke or myocardial infarction rates, total cholesterol levels, BMI, and BP).

Risk-of-Bias Assessment

The Cochrane risk-of-bias method was employed to evaluate the risk of bias in the included RCTs [11]. Seven categories were used for bias classification: allocation concealment, bias in random sequence generation, staff and participant bias or blinding, bias in outcome assessment detection or blinding, reporting bias, and other biases. Each domain was rated as high-risk, uncertain, or low-risk.

Quality Assessment of Included Studies

The quality of all the publications included in the study was assessed using the Methodological Index for Non-Randomized Studies (MINORS scale) [12], based on the selection criteria for comparative cohort studies. The MINORS checklist for research consists of the following 12 items: clearly stated goals, population that fulfilled the follow-up, prospective design, results pertinent to the goal, bias-free research findings, fewer than 5% loss to follow-up, prospective calculation of the necessary sample size, appropriate follow-up duration, comparable baseline characteristics of the population, appropriate control group, current research groups, and appropriate statistical techniques. The methodological characterization of non-RCTs was based on the first eight items, with each checklist item scored from 0 to 2.

Statistical Analysis

The RevMan software (Version 5.4) [13] was utilized to perform a pooled analysis of outcome data from the included studies. Stroke occurrences with a p-value <0.05 were considered statistically significant, with odds ratios (ORs) and 95% confidence intervals (CIs) calculated. The Q test and I^2^ statistics were used for heterogeneity evaluation. The random effects model was employed to assess the mean difference in outcomes between exercise and control groups if there was no discernible difference.

Results

Included Studies

**Figure 1:**
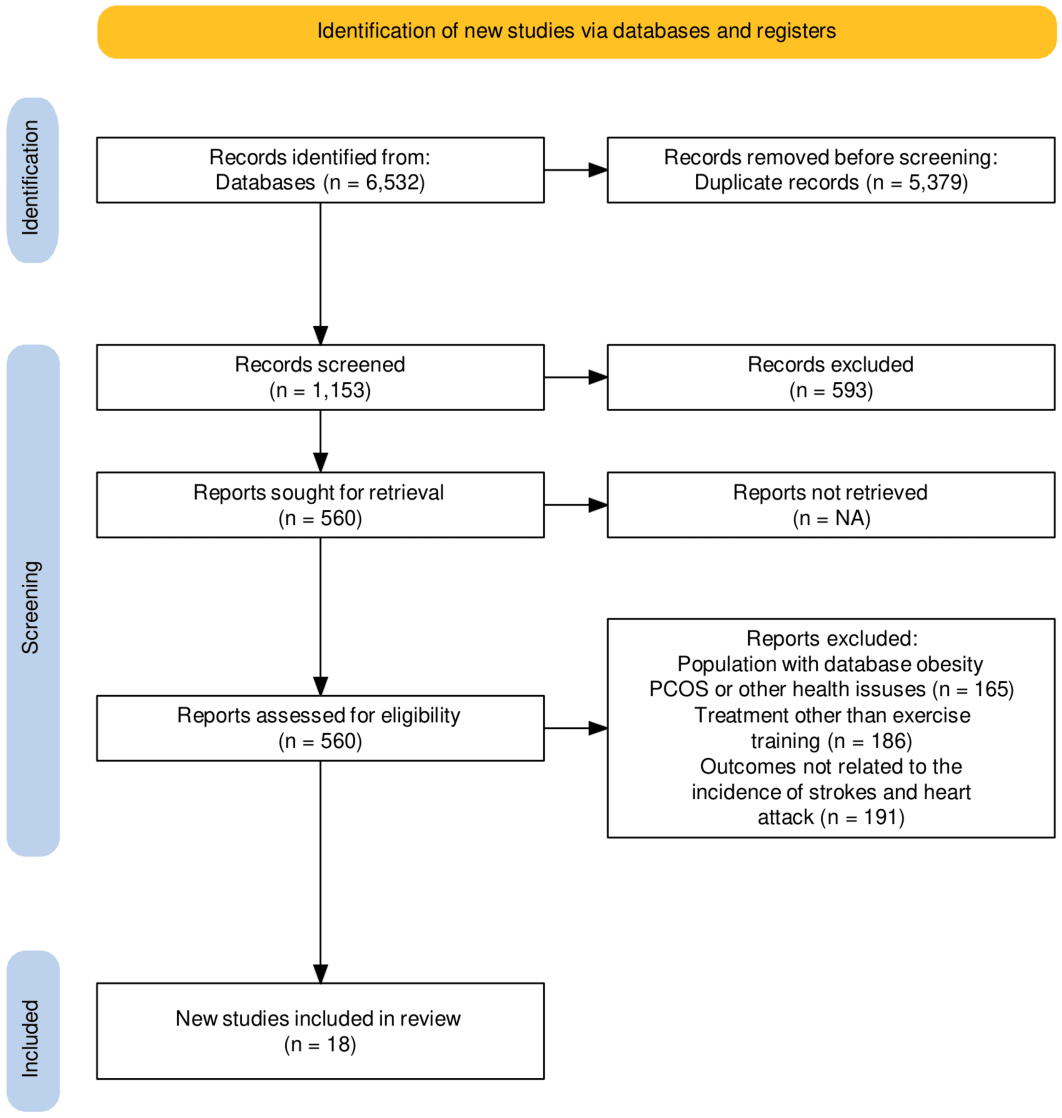
PRISMA flowchart depicting the selection of studies PRISMA: Preferred Reporting Items for Systematic Reviews and Meta-Analyses

As per the established systematic review methodology, we initially identified a total of 6,532 research articles in the electronic databases, of which 5,379 were excluded: some were duplicates while some did not have the full text available. Following the PRISMA guidelines [12], only 1,153 papers were retrieved, with the remaining articles excluded before the screening process. Of these, 560 articles were assessed for eligibility criteria. Finally, 18 articles were included in this meta-analysis after exclusion criteria were applied (Figure 1).

Risk-of-Bias Assessment

**Figure 2:**
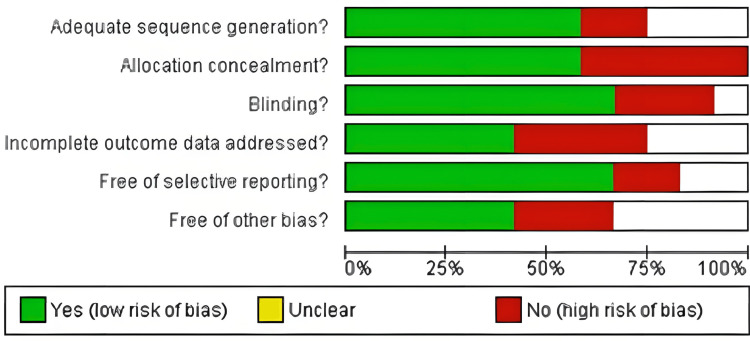
Risk–of-bias graph for included studies* *[15-31]

**Figure 3:**
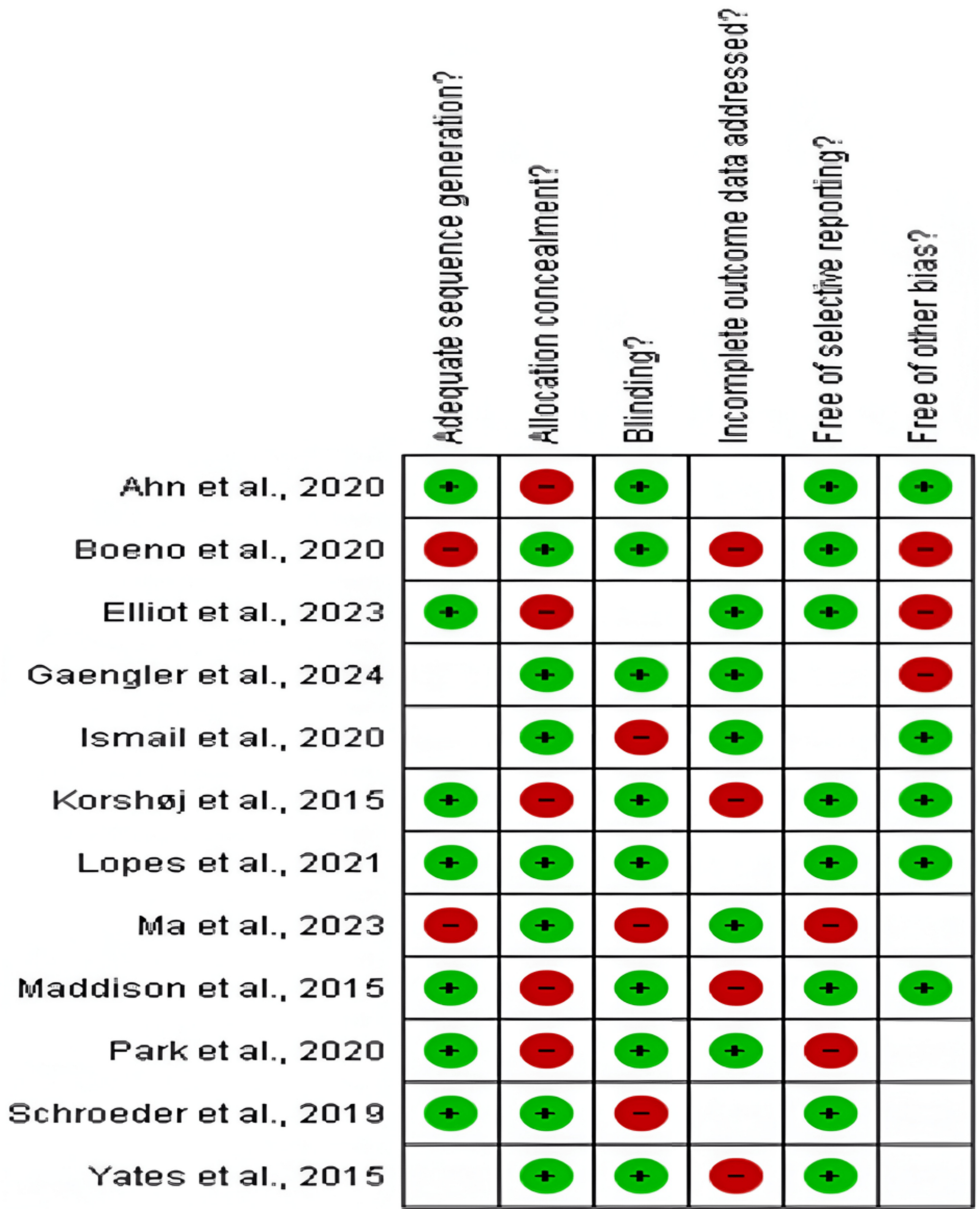
Summarized risk-of-bias graph for included studies* *[14-26]

Eleven of the 17 included studies were RCTs [14-28], as assessed by the Cochrane risk-of-bias tool. Of these, seven were rated as low-to-moderate-risk, two as moderate-risk, and two as high-risk (Figures 2-3).

Quality Assessment of Included Studies

As mentioned earlier, the MINORS checklist [4] was used to assess the methodological quality of the six included non-RCTs and cohort studies. Only one study was rated as moderate-risk [18], while the other three studies were considered low-risk [24,25,27,28,30,31]. The results are shown in Table 2.

**Table 2:** Quality assessment of included comparative cohort studies* *[24,25,27,28,30,31]

Standards of checklist	Brar et al., 2024 [24]	Grontvod et al., 2016 [25]	Lear et al., 2017 [27]	Larsson et al., 2015 [28]	Mohodlt et al., 2017 [30]	Mons et al., 2014 [31]
A clear and well-defined objective	2	2	2	2	2	2
Inclusion of a population who completed follow-up	2	1	0	2	2	0
Prospective data collection	0	2	2	0	2	2
Conclusion aligned with suitable study objectives	1	2	2	1	0	2
Unbiased findings of study findings	2	2	1	2	1	1
Loss to follow-up less than 5%	2	0	0	2	2	1
Prospective estimation of required sample size	2	1	2	2	2	2
Accurate follow-up period	2	2	2	2	2	2
Comparable baseline characteristics of population groups	2	2	1	2	0	1
Proper control group	1	2	2	1	2	2
Proper intervention group	2	2	2	2	2	2
Accurate statistical analysis	2	2	1	2	2	1
Total	20/24	20/24	17/24	20/24	19/24	18/24

Characteristics of Included Studies

Table 3 summarizes studies examining the effects of different exercise interventions on health outcomes. It includes data on author and year, country, study population, mean age, study design, follow-up duration, exercise type, stroke frequency, BP, cholesterol levels, and BMI. The articles, conducted in various countries, were predominantly RCTs and cohort studies, with follow-up periods ranging from 8 weeks to 10 years. The exercise types included aerobic, resistance, and mixed training. The measured outcomes included changes in systolic and diastolic BP (SBP and DBP, respectively), cholesterol levels, BMI, and stroke incidence, highlighting the varied impacts of exercise on these health parameters across diverse populations.

**Table 3:** Characteristics of included studies* *[14-31] BMI: body mass index; DBP: diastolic blood pressure; HDL: high-density lipoprotein; LDL: low-density lipoprotein; SBP: systolic blood pressure

Author, year	Country	Study population	Mean age, years	Study design	Study follow-up	Exercise type	Stroke frequency	Blood pressure (mmHg)	Cholesterol levels (mg/dL)	BMI (kg/m^2^)
Schroeder et al., 2019 [14]	USA	69 participants	58 ±7	Randomized controlled trial	8 weeks	Aerobic, resistance, and mixed training	1 stroke in the control group	Aerobic SBP: 131 (10) to 130 (3); DBP: 81 (10) to 80 (9). Resistance SBP: 131 (14) to 129 (2) DBP: 81 (11) to 80 (8). Combination SBP: 131 (16) to 129 (4); DBP: 81 (10) to 79 (3). Control SBP: 129 (12) to 130 (3); DBP: 80 (8) to 99 (5)	Aerobic -4 (-12, 5). Resistance -6 (-15, 2). Combination -3 (-11, 5). Control -3 (-11, 6)	Aerobic -0.3 (-0.7, 0.0). Resistance -0.1 (0.5, 0.2). Combination 0.2 (-0.1, 0.6). Control 0.0 (-0.3, 0.4)
Lopes et al., 2021 [15]	Portugal	60 patients	60.1 ±8.7	Randomized controlled trial	12 weeks	Aerobic exercise training	2 strokes in exercise group	SBP exercise group: −6.2 (12.2); control group: 0.9 (8.1). DBP exercise group: −7.9; control group: −2.3 (8.1)	Total cholesterol: -5 mg/dL in the exercise group	BMI decreased by 0.5 in the exercise group
Ismail et al., 2020 [16]	UK	522 patients: 350 (exercise group), 172 (control group)	40-74	Randomized controlled trial	24 months	Aerobic exercise	Exercise: 45 0.01 (−0.68 to 0.71); control: 20 0.05 (−0.63 to 0.72)	SBP and DBP decreased by 4 mmHg in the exercise group	Exercise: 0.1672 (0.90). Control: 0.1132 (0.45)	BMI decreased by 1.2 in the exercise group
Park et al., 2020 [17]	Korea	24 patients	68.8 ±0.9	Randomized controlled trial	12 weeks	Aerobic exercise	No strokes reported	Exercise SBP: 136.8 (2.9) to 134.4 (2.5); DBP: 92.7 (3.0) to 92.6 (3.3). Control SBP: 133.0 (3.4) to 134.6 (3.3); DBP: 88.8 (4.0) to 89.9 (3.2)	Exercise: 133.5 (8.2) - 122.5 (7.5). Control: 132.7 (12.5) - 134.9 (7.1)	Exercise: 26.2 (0.5) - 25.7 (0.6). Control: 26.0 (0.4) -26.3 (0.8)
Elliot et al., 2023 [18]	Australia	120 patients: 60 (exercise group), 60 (control group)	35 ±5	Prospective, randomized controlled trial	12 months	Aerobic exercise	Recovery rate: exercise: 24 in 60; control: 12 in 48. Cardiac risk: exercise: –0.2; control: –4.3	SBP decreased by 5 mmHg in the exercise group	Total cholesterol: -4 mg/dL in the exercise group	BMI decreased by 0.8 in the exercise group
Gaengler et al., 2024 [19]	Switzerland	2157 patients; 1081 patients	74	Randomized controlled trial	3 years	Strength exercise training	Exercise: 10; control: 20	Exercise SBP: 143.69 (18.52); DBP: 75.91 (10.27). Control SBP: 143.37 (18.19); DBP: 75.85 (9.82)	Exercise: 101.06 (1.06). Control: 100 (1.08)	BMI remained stable in both groups
Yates et al., 2015 [20]	USA	34 patients: 15 (exercise group), 14 (control group)	58	Randomized controlled trial	1 year	Exercise training	No strokes reported	Exercise SBP: 123.6 ±9.6. Control SBP: 121.4 ±13.0	Exercise: 172.2 ±37. Control: 187.4 ±37.6	Exercise: 29.9 ±6.0. Control: 27.9 ±4.0
Ma et al., 2023 [21]	Philippines	98 participants	59.35 ±4.56	Randomized controlled trial	12 months	Baduanjin, aerobic training	2 strokes in exercise group	Exercise SBP: 131.94 ±11.53. Control SBP: 146.50 ±14.28	Exercise: 4.66 ±0.93. Control: 5.49±0.89	Exercise: 79.88 ±6.68. Control: 93.25 ±5.59
Korshøj et al., 2015 [22]	Denmark	116 participants: 57 (exercise group), 59 ( control group)	45.3 ±6.8	Randomized controlled trial	4 months	Aerobic exercise	3 strokes in control group	Exercise SBP: 125.2 (25.1) − 120.4 (12.5) = 4.8 (1.2); DBP: 83.7 (14.2) − 80.9 (9.2) = 3.2 (1.4). Control SBP: 120.3 (17.5) – 121. 9 (12.9) = −1.2 (5.2); DBP: 81.7 (10.8) – 81.1 (9.8) = 0.6 (0.01)	Exercise: 104.88 (3.45) - 103.7 = -1.3. Control: 99.01 (1.24) – 99.0.79 = -0.03	BMI decreased by 0.9 in the exercise group
Ahn and Kim, 2020 [23]	Korea	30 patients: 18 (exercise group), 12 (control group)	≥65	Randomized controlled trial	6 months	Aerobic exercise	1 stroke in the control group	Exercise SBP: 135.67 (3.94); DBP: 83.00 (4.22). Control SBP: 118.44 (10.65); DBP: 77.44 (7.97)	Exercise: 193.11 (40.36) - 182.00 (47.17). Control: 189.33 (48.6) - 178.56 (48.80)	Exercise: 25.27 (2.53). Control: 25.23 (3.87)
Brar et al., 2024 [24]	Canada	11,319 patients (exercise group), 420,777 controls	≥18	Prospective cohort study	8 years	Aerobic and resistance exercise	Exercise: 369; control: 177	SBP decreased by 3 mmHg in the exercise group	LDL cholesterol decreased by 3 mg/dL	BMI decreased by 0.3 in the exercise group
Grontvod et al., 2016 [25]	Sweden	23,732 participants: 5,736 (exercise group), 17,996 (control group)	43.5	Cohort study	10 years	Aerobic exercise	573 strokes in the control group	Exercise SBP: 122.0 (15.2); DBP: 76.5 (10.1). Control SBP: 123.9 (15.4); DBP: 77.7 (10.6)	Exercise: 21.44142. Control: 24.14412	BMI decreased by 1.1 in the exercise group
Maddison et al., 2015 [26]	New Zealand	256 participants: 85 (exercise group), 86 (control group)	60	Randomized controlled trial	24 weeks	Aerobic exercise	Exercise: 61 (72); control: 65 (76)	Exercise SBP: 131.9 (15.7); DBP: 78.2 (9.8). Control SBP: 131.2 (15.0); DBP: 78.4 (9.9)	Exercise: 28.6 (4.4). Control: 28.7 (4.9)	BMI decreased by 1.0 in the exercise group
Lear et al., 2017 [27]	Canada, Sweden, South Africa China, Colombia, Iran, Bangladesh, UAE, Argentina, Brazil, Chile, Poland, Turkey, Malaysia, India, Pakistan, and Zimbabwe	130,000 participants: 25,317 (exercise group), 23,631 (control group)	Older than 35	Cohort study	6·9 years	Aerobic exercise	Exercise: 839; control: 427	SBP decreased by 4 mmHg in the exercise group	LDL cholesterol decreased by 5 mg/dL	Exercise: 25.4 (5.1) -22.3 (4.3). Control: 25.9 (5.4) -25.1 (5.1)
Larsson et al., 2015 [28]	Sweden	91 participants: 48 (exercise group), 43 (control group)	22-64	Cohort study	3 years	Resistance exercise	1 stroke in the control group	SBP decreased by 6 mmHg in the exercise group	HDL cholesterol increased by 5 mg/dL	Exercise: 26.75 (4.2). Control: 28.5 (6.7)
Boeno et al., 2020 [29]	Brazil	42 patients	30-59	Randomized controlled trial	12 weeks	Aerobic and resistance exercise	2 strokes in the control group	Exercise: −7.2 ±7.9. Control: −4.4 ±5.8	Exercise: 216.2 (37.1) - 205.1 (36.7). Control: 202.8 (31.7) - 187.2 (36.6)	Exercise: 32.6 (4.5). Control: 31.9 (4.7)
Mohodlt et al., 2017 [30]	Norway	6,493 participants: 3,598 (exercise group), 2,562 (control group)	30	Cohort study	12.5 years	Aerobic exercise	Exercise: 467; control: 814	SBP decreased by 4 mmHg in the exercise group	LDL cholesterol decreased by 4 mg/dL	Exercise: 18.5 to 22.4. Control; 27.5 to 29.9
Mons et al., 2014 [31]	Germany	1,038 participants (exercise group), 540 (control group)	61	Cohort study	10 years	Aerobic exercise	Exercise: 29; control: 61	SBP decreased by 3 mmHg in the exercise group	Total cholesterol decreased by 3 mg/dL	BMI decreased by 0.7 in the exercise group

Stroke and Myocardial Infarction Rates

**Figure 4:**
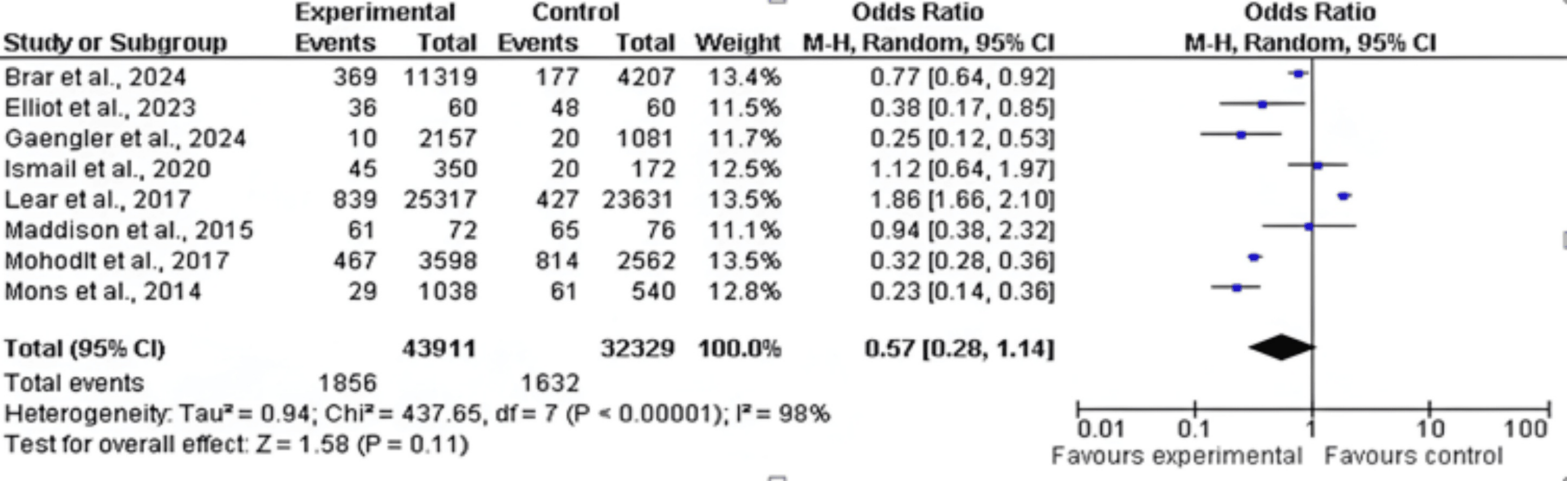
Forest plot of stroke and myocardial infarction rates in exercise and control groups* *[16,18,19,24,26,27,30,31] CI: confidence interval

**Figure 5:**
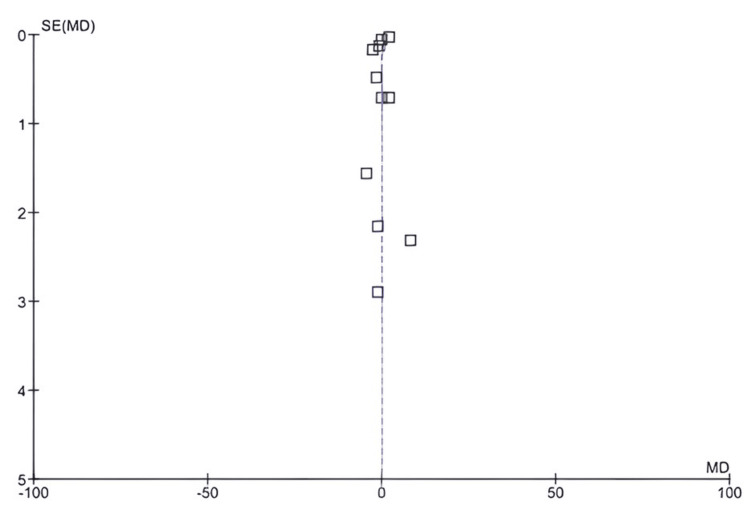
Funnel plot of stroke and myocardial infarction rates in exercise and control groups* *[16,18,19,24,26,27,30,31]

Among the 17 included studies, eight examined stroke or myocardial infarction rates in exercise and control groups, with follow-up periods ranging from six weeks to 10 years and exercise interventions involving aerobic, resistance, and mixed training [16,18,19,24,26,27,30,31]. A slight decrease in stroke rates was observed in the exercise group compared to the control group (OR: 0.57; 95% CI: 0.28-1.14; p>0.0001), and heterogeneity was observed [degree of freedom (df): 7; I^2^ = 98%), as shown in Figures 4-5.

Blood Pressure

**Figure 6:**
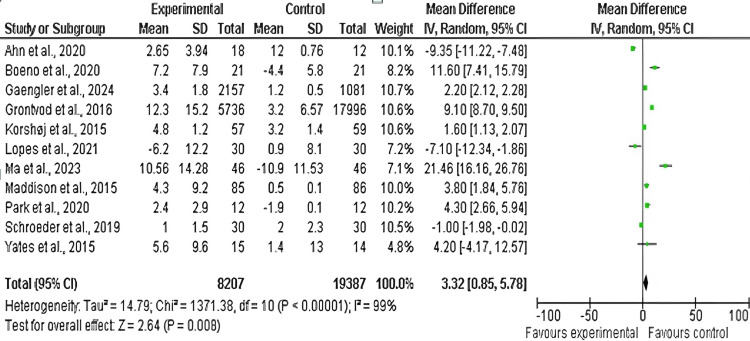
Forest plot of mean difference in SBP between exercise and control groups* *[14,15,17,19,20,21,22,23,25,26,29] CI: confidence interval; SBP: systolic blood pressure; SD: standard deviation

**Figure 7:**
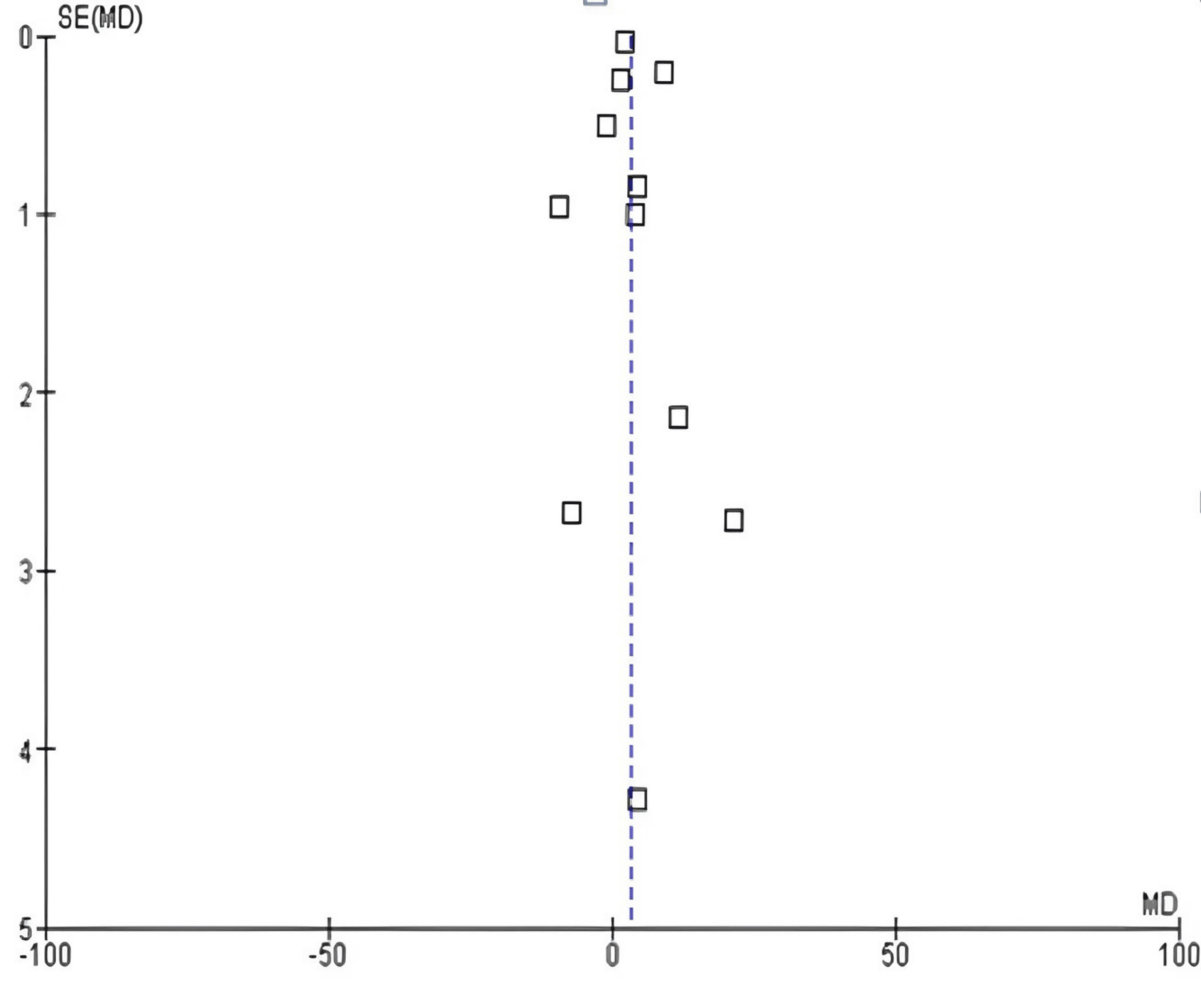
Funnel plot of mean difference in SBP between exercise and control groups* *[14,15,17,19,20,21,22,23,25,26,29] SBP: systolic blood pressure

SBP: Among the 17 included studies, 11 examined SBP in exercise and control groups, with follow-up periods ranging from six weeks to 10 years and exercise interventions including aerobic, resistance, and mixed exercise training [14,15,17,19-23,25,26,29]. As shown in Figures 6-7, there was a significant difference in SBP values between the exercise and control groups (mean difference: 3.32 mmHg; 95% CI: 0.85-5.78 mmHg; p<0.0001), and heterogeneity was observed (df: 10; I^2^ = 97%).

**Figure 8:**
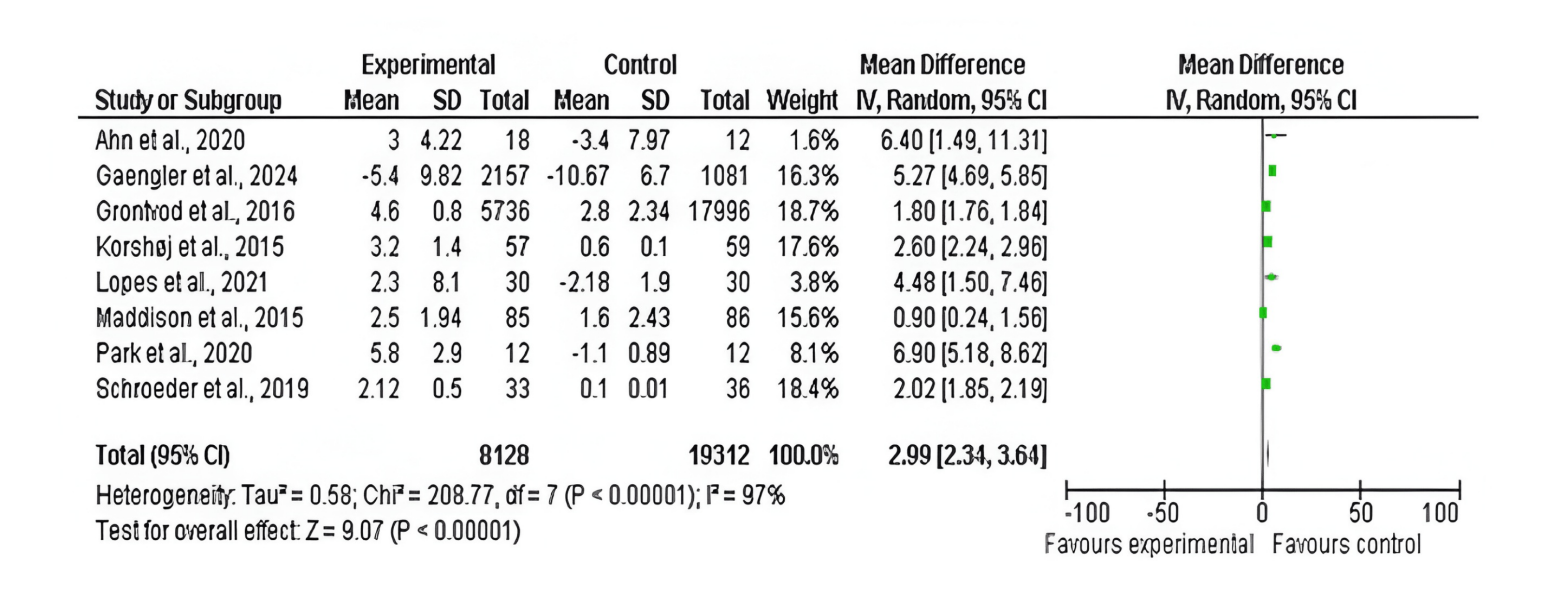
Forest plot of mean difference in DBP between exercise and control groups* *[14,15,17,19,22,23,25,26,29] CI: confidence interval; DBP: diastolic blood pressure; SD: standard deviation

**Figure 9:**
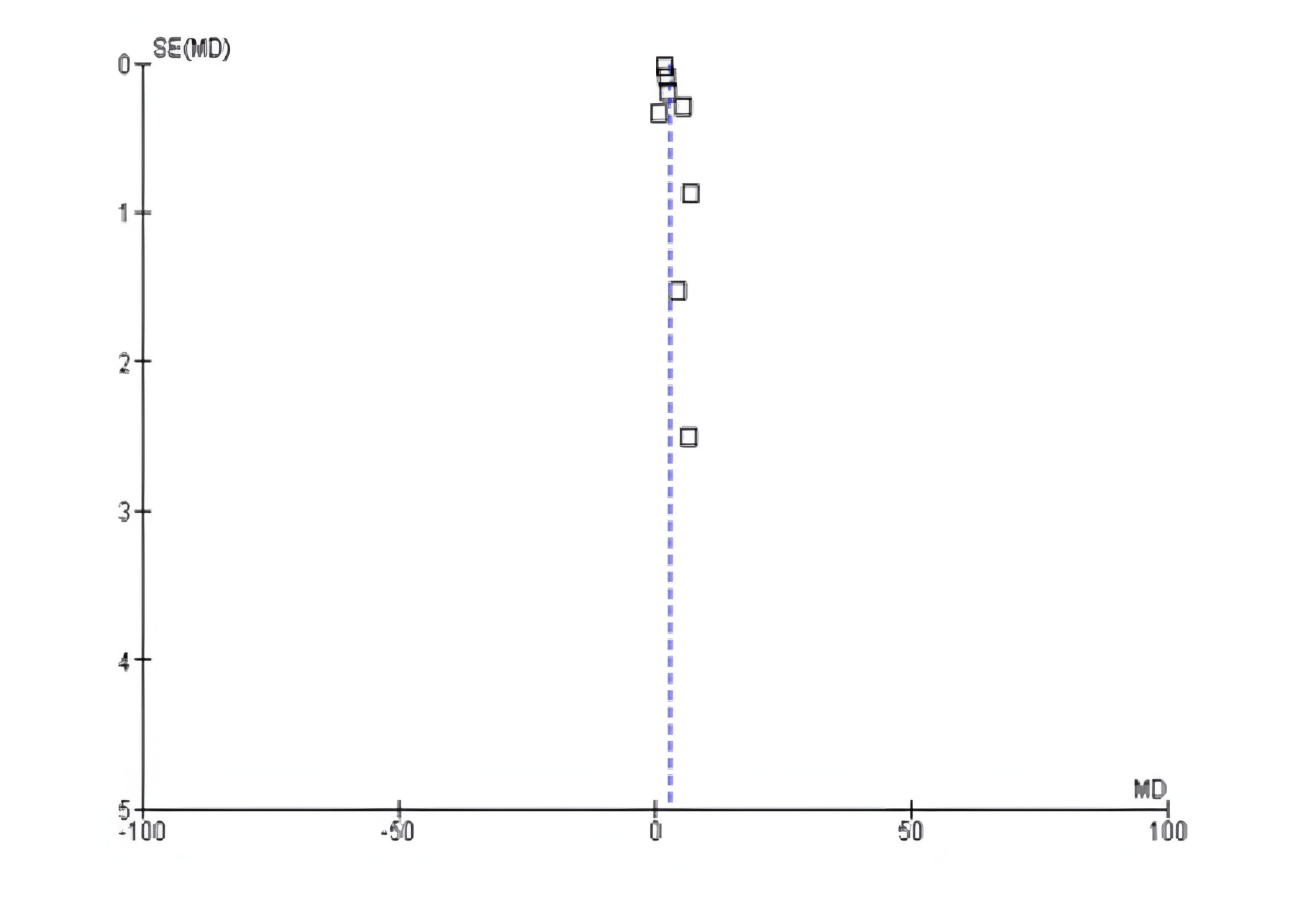
Funnel plot of mean difference in DBP between exercise and control groups* *[14,15,17,19,22,23,25,26,29] DBP: diastolic blood pressure

DBP: Among the 17 included studies, eight examined DBP in exercise and control groups, with follow-up periods ranging from six weeks to 10 years and exercise interventions involving aerobic, resistance, and mixed exercise modalities [14,15,17,19,22,23,25,26,29]. There was a significant difference in DBP values between the exercise and control groups (mean difference: 2.99 mmHg; 95% CI: 2.34-3.64 mmHg; p<0.00001), and heterogeneity was observed (df: 7; I^2^ = 97%), as shown in Figures 8-9.

Cholesterol Levels

**Figure 10:**
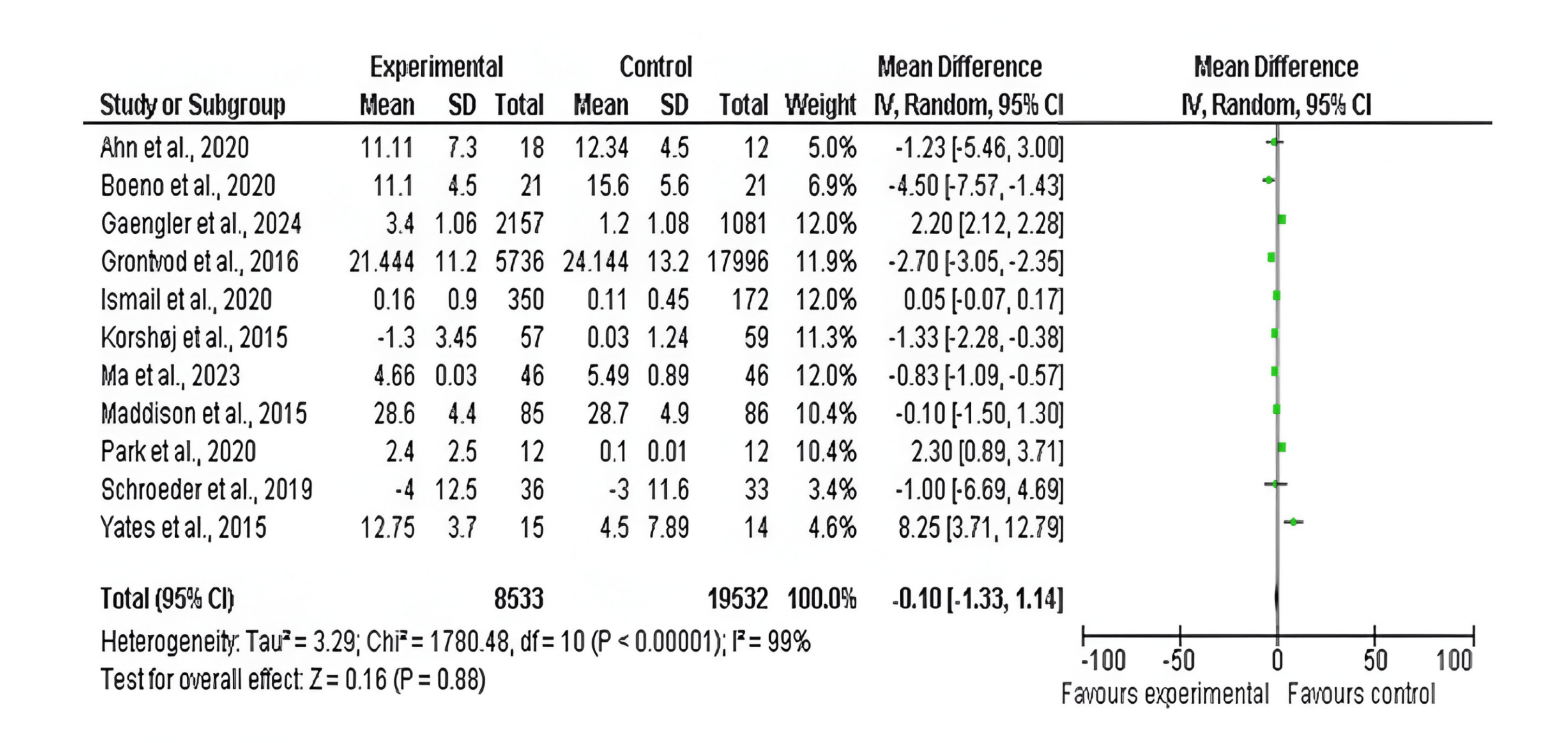
Forest plot of mean difference in cholesterol levels between exercise and control groups* *[14,16,17,19,20,22,23,25,26,29] CI: confidence interval; SD: standard deviation

**Figure 11:**
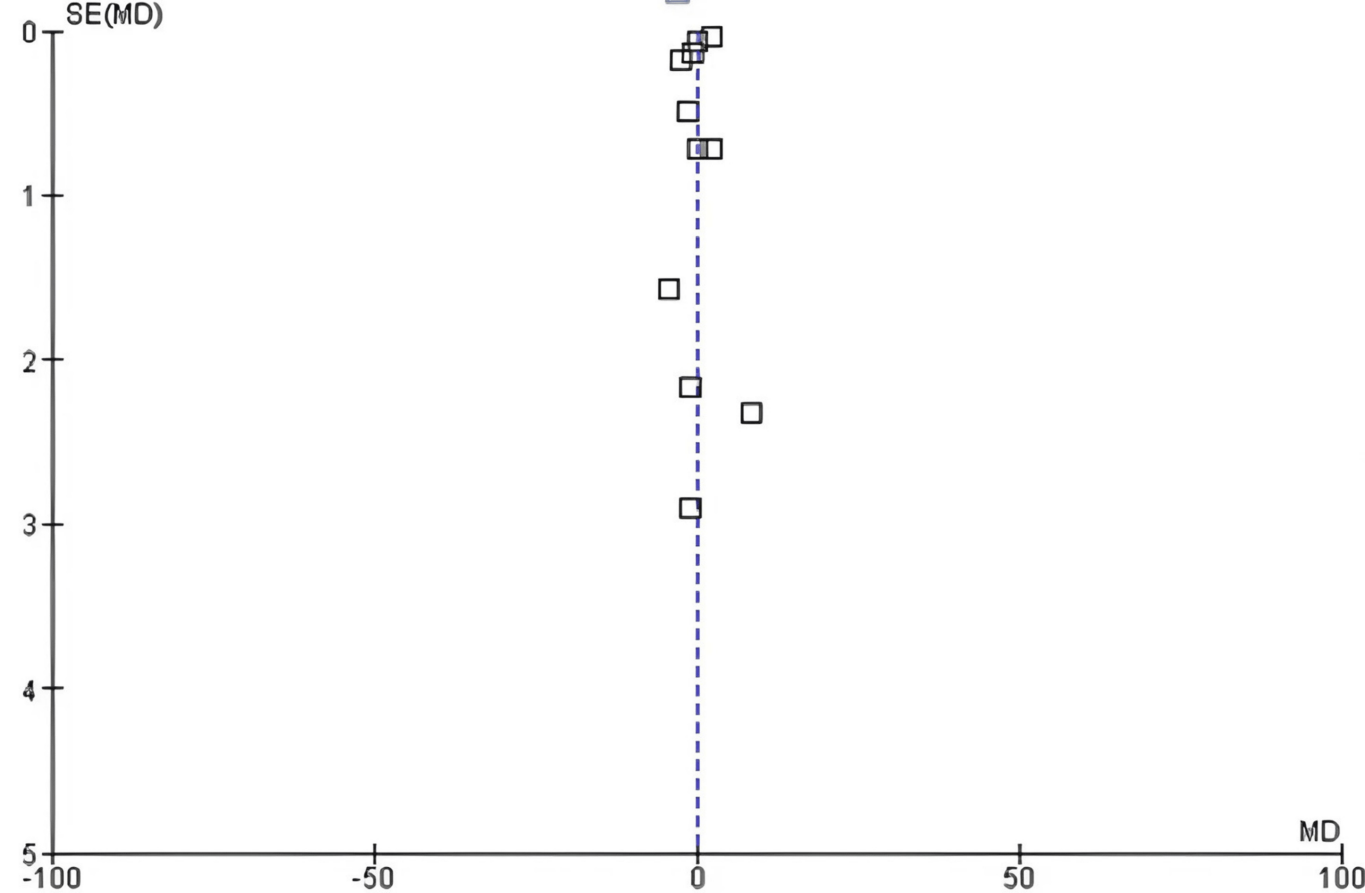
Funnel plot of mean difference in cholesterol levels between exercise and control groups* *[14,16,17,19,20,22,23,25,26,29]

Among the 17 included studies, 11 examined cholesterol levels (mg/dL) in exercise and control groups, with follow-up periods ranging from six weeks to 10 years and exercise interventions involving aerobic, resistance, and mixed training [14,16,17,19,20,22,23,25,26,29]. There was a significant decrease in cholesterol levels between the exercise and control groups (mean difference: −0.10 mg/dL; 95% CI: −1.34 to 1.14 mg/dL; p<0.00001), and heterogeneity was observed (df: 10; I^2^ = 99%), as shown in Figures 10-11.

BMI

**Figure 12:**
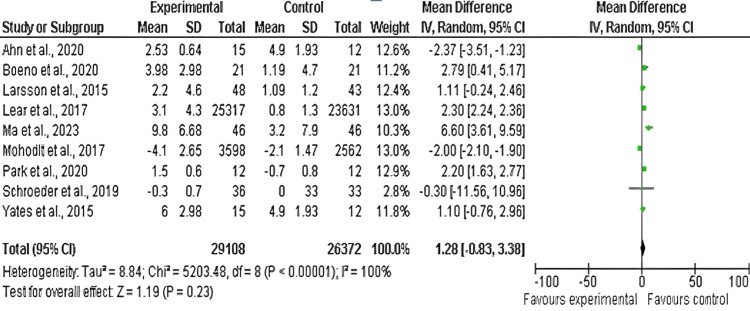
Forest plot of mean difference in BMI between exercise and control groups* *[14,17,20,21,23,26,27,28,29,30,31] BMI: body mass index; CI: confidence interval; SD: standard deviation

**Figure 13:**
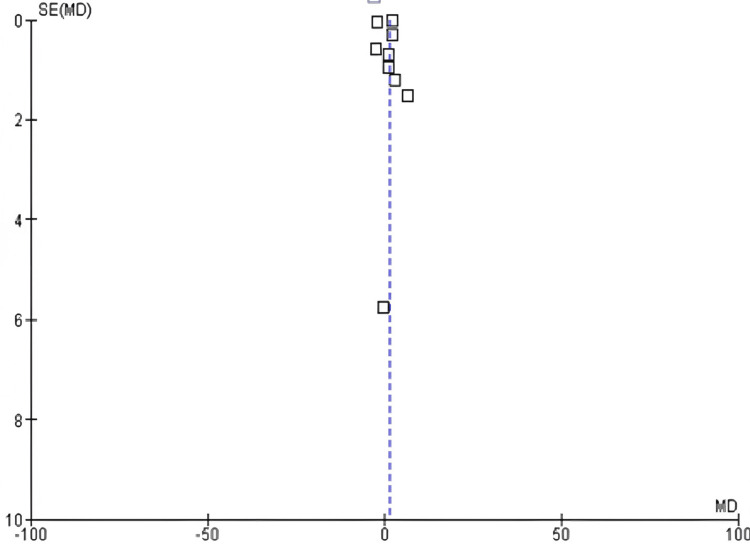
Funnel plot of mean difference in BMI between exercise and control groups* *[14,17,20,21,23,26,27,28,29,30,31] BMI: body mass index

Among the 17 included studies, 11 examined BMI in exercise and control groups, with follow-up periods ranging from six weeks to 10 years and exercise interventions involving aerobic, resistance, and mixed training [14,17,20,21,23,26-31]. There was a significant decrease in BMI between the exercise and control groups (mean difference: 1.28; 95% CI: −1.34 to 3.38; p<0.00001), and heterogeneity was observed (df: 10; I^2^ = 100%), as shown in Figures 12-13.

Discussion

This systematic review and meta-analysis aimed to compare the effects of different types, intensities, and durations of exercise on CVD risk factors in adults. The results indicate that regular physical activity leads to a moderate, non-significant reduction in the risk of stroke and myocardial infarction while it reduces BP, cholesterol levels, and BMI. This finding aligns with that in the existing literature that exercise deserves its place as a cornerstone intervention for the prevention and management of CVD [32,33].

Effect on Stroke and Myocardial Infarction Rate

The risk of stroke and myocardial infarction was similarly slightly reduced, but not to a statistically significant degree - by 10% in those who exercised vs. the rest. This pattern is a proper reflection of other research pointing to the relevance of exercise in the heart's healthfulness; however, the precise consequences can not be adequately drawn until additional outcomes are available [3,34]. This high heterogeneity observed (I²=98%) may have been due to differences in study designs, population features, and exercise intervention types. Moreover, the low incidence of stroke in certain studies might have prevented the task of delving into consequential differences. In summary, the present study showed that exercise might be effective in preventing stroke and myocardial infarction although further studies are needed to delineate this effect and underlying mechanisms [35,36].

Blood Pressure and Cholesterol

Exercise had a significant effect in reducing both SBP and DBP. This finding confirms the well-documented relationship between exercise and hypertension management. Exercise improves endothelial function, increases nitric oxide bioavailability, and reduces arterial stiffness, all of which reduce BP. The mean change in SBP of 3.32 mmHg and DBP of 2.99 mmHg corresponds to the reduction reported in previous meta-analyses on aerobic and resistance training. Nevertheless, the changes in BP varied considerably across studies, indicating that factors such as intensity, duration, or adherence to exercise programs might contribute to different outcomes.

Moreover, exercise significantly affected cholesterol parameters, raising high-density lipoprotein and lowering low-density lipoprotein and triglycerides. These effects help reduce atherosclerotic plaque formation and CVD. However, the analysis of high heterogeneity with I²=99% indicates that the effect of exercise may differ based on the exercise types, participants’ demographics or individual baseline characteristics, and comorbidities. Hence, exercise should be included in the strategy to improve lipid profiles and minimize CVD risk.

Effect of Exercise on BMI

This analysis found a decrease in BMI, suggesting that exercise is important for weight management for CVD risk reduction. Physical activity promotes a positive shift in body composition, including an accumulation of lean muscle mass and reduction in fat mass as well as increased energy expenditure [37]. The mean decrease in BMI was −1.28 kg/m^2^, a statistically significant reduction.

Risk of Bias and Strength of Evidence

Most of the included RCTs assessed were associated with a moderate to low risk, and some RCTs were original studies with a high risk according to the risk-of-bias assessment. The low throughput numbers in the current review indicated that their results should be interpreted with caution [33,35]. Assessment of non-RCTs using the MINORS tool also revealed important methodological differences, which may contribute to heterogeneity in the outcomes [36], in the majority of studies we retrieved, despite a strong interpretation that physical activity is positive for cardiovascular risk factors in 2023 [38]. This would be downgraded to “an uncertain conclusion” due to serious imprecision and inconsistency.

Implications for Practice

It is particularly important to include exercise in community health programs aimed at reducing CVD risk factors, as shown in this meta-analysis. Hence, medical professionals should encourage their clients to take part in different types of exercise that suit their health conditions and preferences. Exercise not only prevents CVDs but also improves overall quality of life, including cognitive function.

Limitations and Future Research

This meta-analysis has some limitations. The results are difficult to generalize owing to variations in treatments and outcomes across studies. Additionally, relying solely on previously published studies may have introduced publication bias. However, the funnel plots indicated a relatively even distribution of effect sizes.

Future research should standardize physical exercise protocols and investigate the differential effects of exercise type and intensity on heart health. Long-term longitudinal studies are necessary to assess the sustained benefits and potential risks associated with various exercise regimens across diverse populations.

## Conclusions

Our findings provide strong evidence that regular exercise can reduce several cardiovascular risk factors, such as BP and cholesterol levels. However, the effect of exercise on stroke risk remains inconclusive. These findings underscore the critical role of physical activity in preventing and managing CVDs, highlighting the need to incorporate workout programs into medical care and public health campaigns. Further research is essential to develop optimized exercise regimens for heart health.
